# Genotype-phenotype spectrum and prognosis of early-onset Marfan syndrome

**DOI:** 10.1186/s12887-023-04357-8

**Published:** 2023-10-28

**Authors:** Aurelija Kemezyte, Ruta Gegieckiene, Birute Burnyte

**Affiliations:** 1 Faculty of Medicine, Vilnius University, M.K. Ciurlionio st. 21, Vilnius, Lithuania; 2 Center of Cardiothoracic Surgery, Clinic of Cardiovascular Diseases, Institute of Clinical Medicine, Faculty of Medicine, Vilnius University, Santariskiu St. 2, Vilnius, Lithuania; 3 Institute of Biomedical Sciences, Faculty of Medicine, Vilnius University, Santariskiu st. 2, LT-08661 Vilnius, Lithuania

**Keywords:** Early-onset Marfan Syndrome, *FBN1*, Exons 24–32, Genotype-phenotype correlations, Skeletal features, Cardiac intervention

## Abstract

**Background:**

Marfan syndrome is a genetic connective tissue disorder affecting skeletal, ocular, and cardiovascular organ systems. Previous research found that pathogenic variants clustered in exons 24–32 of fibrillin-1 (*FBN1*) gene result in more severe clinical phenotypes. Furthermore, genotype-phenotype correlation studies suggested that more severe cardiovascular phenotypes were related to variants held responsible for haploinsufficiency. Our objective was to analyze the differences in clinical manifestations and genotypes of individuals with early-onset Marfan syndrome and to assess their impact on management strategies.

**Methods:**

We analyzed clinical and genetic data of a new patient with early-onset Marfan syndrome together with 51 previously reported ones in the PubMed database between 1991 and 2022.

**Results:**

Analysis showed 94% (49/52) of pathogenic variants clustered in exons 24–32 of the *FBN1*. The most common skeletal features were arachnodactyly (98%), reduced elbow extension (48%), pectus deformity (40%), and scoliosis (39%). Haploinsufficiency variants were reported as having poor outcome in 87.5% of the cases. Among patients carrying variants that substitute a cysteine for another amino acid and those that do not change cysteine content, cardiac intervention was found to be associated with a better outcome (p = 0.035 vs. p = 0.002). Variants that create an extra cysteine residue were found to be associated with a higher risk of ectopia lentis. Additionally, children up to 36-months-old were more often reported as still alive at the time of publication compared to newborns (p < 0.01).

**Conclusions:**

Our findings have implications for prognosis, because different genotype groups and their resulting phenotype may require personalized care and management.

**Supplementary Information:**

The online version contains supplementary material available at 10.1186/s12887-023-04357-8.

## Background

Marfan syndrome (MIM 154,700, MFS) is a hereditary disorder originally reported by professor of paediatrics Antonin-Bernard Marfan (1858–1942). Physician prompted later interest of scientists in this disease, who recognized its Mendelian dominant trait and by 1991 it was confirmed genetically by Dietz et al. in two unrelated patients with missense variants in fibrillin-1 (*FBN1*) gene [1]. *FBN1* itself is a large gene of 230 kb and is fragmented into 65 exons. Pathogenic variants of *FBN1* vary in phenotypes, which differently affect tissue damage severity and timing of disease manifestation. Major manifestations are known as ectopia lentis, aortic root dilation, and dural ectasia, together with other skeletal features, such as wrist and thumb sign, pectus deformity, and others. Hence, diagnostic criteria or revised Ghent nosology, which consists of those features, is used worldwide to diagnose MFS [2]. However, it does not necessarily fit in the picture of the most robust genotype-phenotype or early-onset form of MFS (EOMFS), also named neonatal MFS (nMFS), which causative mutations are usually located in exons 24–32 [3]. The incidence of classic Marfan’s syndrome is about 2–3 per 10 000 individuals, while the frequency of nMFS is not reported in the literature [4]. In addition, it is difficult to draw a clear line between neonatal and EOMFS, because more clinical phenotypes may exist (further on term “early-onset MFS” will be used for simplicity purpose). To diagnose nMFS, distinct findings, such as mitral or tricuspid valvular insufficiency, pulmonary emphysema, joint contractures, crumpled ears and loose skin were needed to be present together at birth or within first 3 months of life [5], as described by Booms et al. [6]. However, in 2016 Maeda et al. [7] described case series almost corresponding to nMFS, but named it EOMFS, as pulmonary emphysema was not present. Authors suggested that a broader phenotype spectrum of EOMFS may exist without fulfilling Hennekam’s strict definition and noted the importance of elucidating genotype-phenotype correlations in patients with pathogenic variants in this critical region. Life-threatening cardiovascular complications such as aortic root dilation, mitral valve prolapse and regurgitation are frequently found in EOMFS and may not be present together, but are usually associated with poor outcomes [8]. In the past years, a few studies on characterization of *FBN1* pathogenic variants by their genotype-phenotype correlations were reported [9–12]. More detailed research focused on dominant negative effect, associated with in-frame pathogenic variants’ phenotype and haploinsufficiency model, related to premature termination codon variants and analysed a large population of MFS patients, including systematic familial screening to limit referral bias [12]. Authors confirmed that lifelong aortic event risk was strongly associated with premature termination codon pathogenic variants (83%), in-frame pathogenic variants leading to a cysteine loss at the protein level (73%), and variants that do not change the cysteine content at the protein level (61%). Importantly, pathogenic variants leading to an additional cysteine residue had mild risk for aforementioned event (29%). Data on genotype-phenotype correlations are crucial for the early management of MFS.

Here we report a new patient with an EOMFS and a review of the prevalence of clinical characteristics and their manifestation within genotype groups of EOMFS cases published in the literature between 1991 and 2022. Additionally, we were interested in determining how genotyping might be beneficial in a clinical setting for EOMFS patients.

## Methods

### Patients’ population

The study population consisted of one novel patient and 51 individual cases identified in the literature with a heterozygous likely pathogenic or pathogenic variant of the *FBN1* gene, which resulted in EOMFS. Written informed consent for participation in this study was obtained from patient parents before publishing this article. Data were collected using standardized form created based on revised Ghent nosology by Loeys et al. [2]. Collected data included demographics, age at examination, genetic profile, musculoskeletal, ophthalmologic, cardiovascular features, intervention, and outcomes.

### Literature review

We searched PubMed database utilizing EOMFS-related keywords and a query “(((Marfan syndrome) AND (((((Early-onset) OR (Newborn)) OR (Toddler)) OR (Infant)) OR (Neonatal)))” with limits activated (1991–2023, English). 632 articles were identified. Two independent researchers reviewed those manuscripts by title and abstract excluding non relevant articles. 201 manuscripts were selected for further investigation and fully revised. The exclusion criteria were (1) insufficient molecular genetic data about the case (2) genetically not confirmed cases (3) prenatally diagnosed cases (4) cases without full clinical information needed (5) cases with variants now classified as likely benign (6) patients with positive family history. To elucidate some of the exclusion criteria, we want to specify that prenatal MFS cases were not included as they are evaluated during sonoscopy study, which does not provide clinical features for diagnosis and can be extremely inaccurate, while the patients with a positive family history where not included in this study, because probands usually have more severe phenotype than their family members, which could mislead us in evaluating the severity of EOMFS clinical features. After ultimate revision, thirty-eight articles were included in the final analysis.

### Genotype related categorization

In this study, we divided 52 individuals into two major groups: haploinsufficiency and a dominant negative effect group (DN). Pathogenic variants affecting cysteine content and those not affecting cysteine content were assigned to the DN group and others to the haploinsufficiency group. Dominant negative effect sample was further divided into three different categories: cys+ (missense variants that substitute for a cysteine), cys– (variants that substitute a cysteine for another amino acid), and cys-no (variants that do not modify cysteine content) group. This categorization strategy was adapted from the methodology created by Arnaud et al., 2021 and is detailed in Supplementary Table 1. Current criteria and guidelines as published by the American College of Medical Genetics and Genomics (ACMG) and the Association for Molecular Pathology (AMP) were followed to evaluate variant pathogenicity [13]. A freely available online software tool that implements these criteria was used for the revision of all identified variants [14].

### Statistical analysis

Statistical analysis was conducted using SPSS Statistics V.20.0 (IBM). Descriptive statistics of patients’ clinical characteristics are reported in frequencies and percentages (N, %). Quantitative data are expressed as median (quartiles). Qualitative variable features within groups were analysed using the chi-square test of independence, which results were considered statistically significant when p < 0.05.

## Results

### Case report

**Fig. 1:**
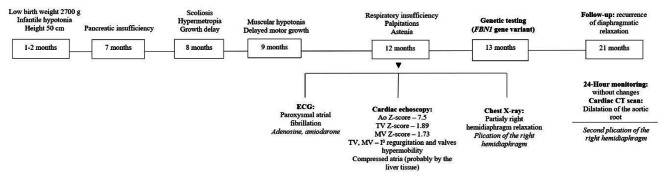
Timeline of the developmental and diagnostic milestones

**Fig. 2:**
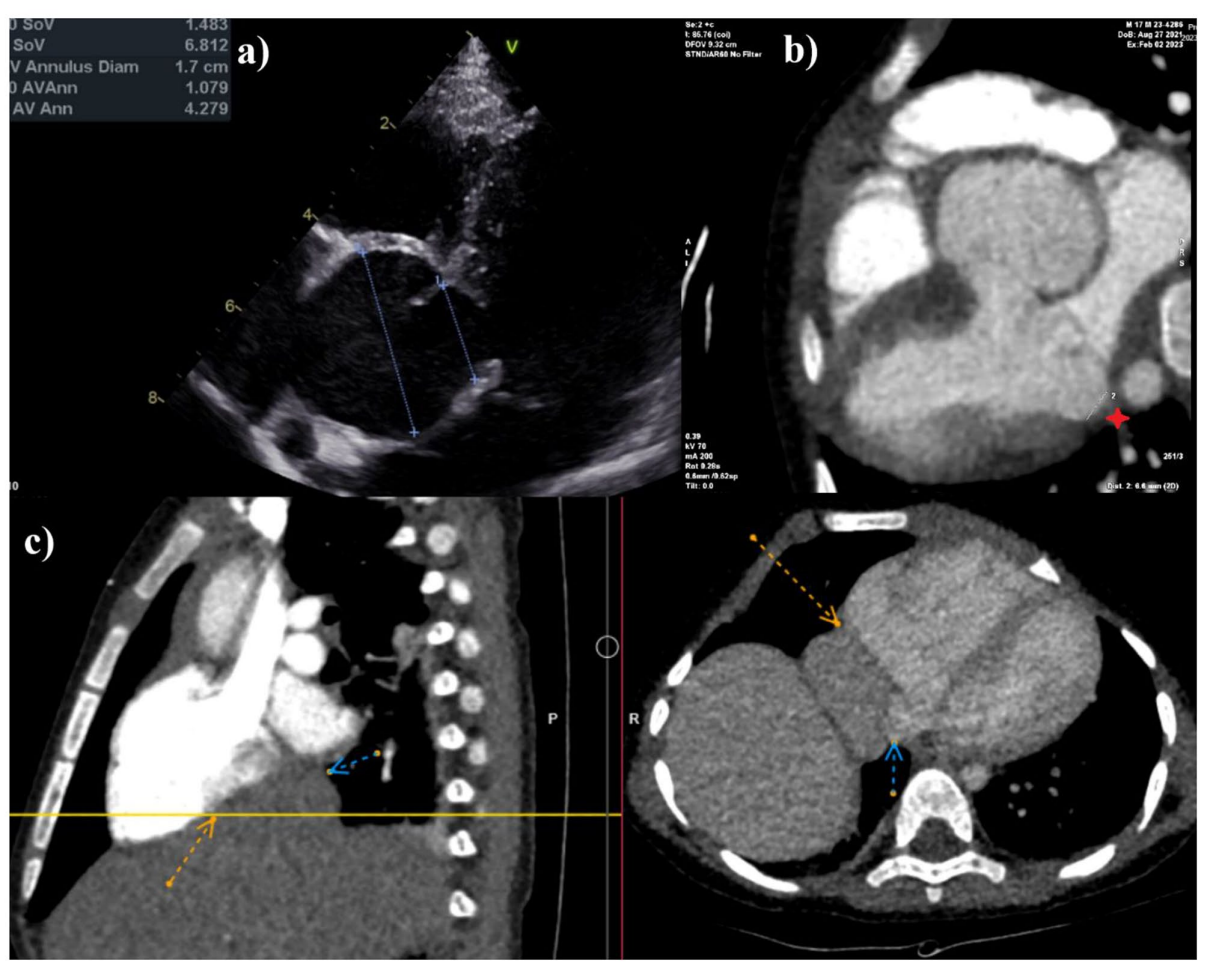
(**a**) Echocardiogram illustrating dilatation of the aortic root (sinuses of Valsalva); (**b**) Transversal computer tomography (CT) illustrating mitral annular disjunction distance (6.6 mm); (**c**) Sagittal and transversal computer tomography (CT) illustrating diaphragmatic relaxation. Left liver lobe entering the mediastinum

A male patient, first child of a previously healthy family, was born full term with weight and height of 2700 g (5–10°percentile) and 50 cm (50° percentile), respectively (Fig. 1). He presented infantile hypotonia and a poor weight gain. At age of 7 months, pancreatic insufficiency was confirmed and a continuous pancrelipase therapy started. At age of 8 months, he developed left spinal deviation and low degree hypermetropia. On physical examination characteristic facial features included enophthalmos, malar hypoplasia, and microretrognathia. Musculoskeletal system was noteworthy for chest asymmetry, pectus carinatum, and scoliosis. Additionally, the patient appeared pale and had arachnodactyly. At age of 1, he was hospitalized due to the shortness of breath. Paroxysmal supraventricular tachycardia was registered on 12-lead electrocardiogram (ECG). Adenosine was ineffective. Amiodarone was used to terminate arrhythmia. Chest X-ray showed relaxation of right hemidiaphragm. Abdominal ultrasonography was done without any signs of hepatomegaly. Echocardiogram showed that left and right atriums were compressed by the liver tissue, mitral and tricuspid valve prolapse with mild to moderate degree regurgitation. It also revealed markedly dilated aortic root (3.1 cm; Z score + 7.5) (Fig. 2. a)). Echocardiogram findings were confirmed by computerized tomography (CT) angiogram with the calculations of mitral anullar disjuntion distance – 6.6 mm (Fig. 2. b-c). In consequence, diaphragm plication was performed twice in 9-month period. Follow-up during this period was without any events of arrhythmia and no significant changes in echocardiogram evaluations. Blood test results during follow-up were normal. No cardiac interventions were performed. The patient is on low dose of beta blockers and amiodarone, and a follow up every 2–4 months.

Whole exome sequencing data revealed a de novo heterozygous pathogenic missense variant c.3661T > C, p.(Cys1221Arg) of *FBN1* in exon 30. This variant has already been reported in the literature as causing EOMFS [15].

### Patient’s population

A total of 52 cases along with our patient were included in the final study [6–8, 15–49]. The summary of their demographic and genetic profile characteristics is outlined in Table 1. The percentage of males was slightly higher than that of females (52% vs. 46%), and gender was unknown for one individual (2%). Age at diagnosis ranged from preterm to infantile children, while full term newborns accounted for 62% (32/52), preterm newborns for 13% (7/52), and the median age at diagnosis of other 13 patients was 8 months (Q1-Q3, 4–11). A significant number of newborns died (73.7%), while older children (30.8%) were alive at the time when the article was published (p < 0.010). Patients reported alive at the time when the article was published had the median age of 60 months (Q1-Q3, 14–120) and the oldest patient reported alive was 23-year-old. For chi-square test newborns and preterm newborns were placed in one group and compared with infants, which exact age is specified in Table 1.

**Table 1:** Demographic and genetic profile characteristics. Number (N); not applicable (NA); variants that substitute a cysteine for another amino acid (Cys–); missense variants that substitute for a cysteine (Cys+); variants that do not modify cysteine content (Cys-no)

		Gender				Age at examination			Genetic profile					
		N, (%)	Female	Male	NA	Newborn	Preterm	Infants, months (mean, Q1-Q3)	Exon 24–32	De novo	Missense	Deletion	Splice site	Insertion
**All**		52 (100)	24 (46)	27 (52)	1 (2)	32 (62)	7 (13)	10.9 (Q1-Q3, 4–11)	49 (94)	48 (92)	39 (75)	6 (12)	6 (12)	1 (2)
**Haploinsufficiency**		8 (15)	3 (37.5)	5 (62.5)	0 (0)	7 (87.5)	1 (12.5)		7 (87.5)	6 (75)	0 (0)	2 (25)	6 (75)	
**Dominant negative effect**	Cys–	18 (35)	9 (50)	8 (44)	1 (6)	7 (39)	5 (28)	6.6 (Q1-Q3, 4–9)	17 (94)	18 (100)	15 (83)	3 (17)		
	Cys+	3 (6)	2 (67)	1 (33)		2 (67)		36	3 (100)	3 (100)	2 (67)			1 (33)
	Cys-no	23 (44)	10 (43.5)	13 (56.5)		16 (70)	1 (4)	10.9 (Q1-Q3, 2–18)	22 (96)	21 (91)	22 (96)	1 (4)		

### **FBN1** gene variants and genotype groups demographics

Majority (94%) of the pathogenic variants in the *FBN1* gene were clustered in exons 24–32 (Table 1). Of the 52 cases included in the study, almost all of the variants (48/52, 92%) were *de novo*, while four lacked segregation data on family history (4/52, 8%). Missense variants were the most common substitution (75%), followed by exon deletions and splice site variants (12%), with the only one insertion present (2%). After categorizing the patients into groups based on their genotype, cys-no was the largest group (23/52, 44%), followed by cys– (18/52, 35%), haploinsufficiency (8/52, 15%) and cys+ (3/52, 6%).

### Clinical characteristics

Clinical characteristics distribution within groups and overall features are highlighted in Table 2. Commonly observed clinical characteristics were arachnodactyly (98%), mitral regurgitation/prolapse (96%), and aortic root dilation (90%). To begin with skeletal system, reduced elbow extension was most frequently reported (6/8, 75%) in the haploinsufficiency group and similarly distributed within cys–, cys + and cys-no groups, 44% vs. 33% vs. 43.5%, respectively. Quite large sample of patients had pectus deformity (21/52, 40%), which largest percentage was present in cys + group (100%), similar proportion in haploinsufficiency and cys-no groups (37.5% vs. 39%), and the smallest in cys– group patients (16.7%). Scoliosis or thoracolumbar kyphosis was present in almost half of the patients (39%) and similarly distributed between groups, except for a haploinsufficiency group, where it was less commonly reported (1/8, 12.5%).

**Table 2:** Distribution of clinical features within genotype groups. Number (N); not applicable (NA); variants that substitute a cysteine for another amino acid (Cys–); missense variants that substitute for a cysteine (Cys+); variants that do not modify cysteine content (Cys-no)

		N, (%)	Arachnodactyly	Camptodactyly	Wrist sign	Thumb sign	Pectus deformity	Talipes	Plain pes planus	Hindfoot deformity	Scoliosis/kyphosis	Reduced elbow extension	Senile face appearance	Dolichocephaly	Enophtalmos	Downslanting palpebral fissures	Malar hypoplasia	Micrognatia	Retrognathia	Lens subluxation	Ectopia lentis	Mitral valve regurgitation/prolapse	Aortic root dilation	Diaphragm herniation/relaxation	Cardiac intervention	Outcome (death)
All (%)		52 (100)	51 (98)	5 (10)	2 (4)	5 (10)	21 (40)	1 (2)	9 (17)	4 (8)	20 (39)	25 (48)	11 (2)	16 (31)	13 (25)	19 (37)	2 (4)	12 (23)	10 (19)	6 (12)	15 (29)	50 (96)	47 (90)	9 (17)	21 (40)	32 (62)
Haploinsufficiency group		8 (15)	8 (100)	2 (25)	0 (0)	0 (0)	3 (37.5)	0 (0)	4 (50)	0 (0)	1 (12.5)	6 (75)	2 (25)	2 (25)	2 (25)	4 (50)	1 (12.5)	0 (0)	1 (12.5)	1 (12.5)	2 (25)	8 (100)	7 (87.5)	1 (12.5)	2 (25)	7 (87.5)
Dominant negative effect group	Cys-	18 (35)	18 (100)	3 (17)	0 (0)	1 (6)	3 (16.7)	0 (0)	2 (11)	1 (6)	7 (39)	8 (44)	4 (22)	7 (39)	2 (11)	8 (44)	0 (0)	5 (28)	4 (22)	2 (11)	5 (28)	18 (100)	16 (89)	3 (17)	7 (39)	11 (61)
	Cys+	3 (6)	3 (100)	0 (0)	1 (33)	1 (33)	3 (100)	0 (0)	1 (33)	1 (33)	1 (33)	1 (33)	1 (33)	1 (33)	1 (33)	0 (0)	1 (33)	1 (33)	1 (33)	0 (0)	2 (67)	3 (100)	3 (100)	0 (0)	1 (33)	1 (33)
	Cys-no	23 (44)	22 (96)	1 (4)	1 (4)	3 (13)	9 (39)	1 (4)	3 (13)	2 (9)	11 (48)	10 (43.5)	4 (17)	6 (26)	8 (35)	7 (30)	0 (0)	6 (26)	4 (17)	3 (13)	6 (26)	21 (91)	20 (87)	5 (22)	10 (44)	13 (57)

As with facial features, downslaping palpebral fissures were observed in 19 cases (37%), dolichocephaly in 16 patients (31%), and enophthalmos in 13 patients (25%). Micrognathia and retrognathia were reported less frequently, with percentages of 23% and 19%, respectively.

The most common ophthalmologic feature was ectopia lentis (29%), which was similarly distributed in the haploinsufficiency, cys–, and cys-no groups (25%, 28%, and 26%, respectively). The highest proportion of ectopia lentis was reported in the cys + group (2/3, 67%).

### Cardiovascular features

Aortic root dilation and mitral regurgitation/prolapse were most frequently observed cardiovascular features (90% and 96%). Mitral regurgitation/prolapse was reported in all haploinsufficiency, cys–, cys + patients, and in 91% of cys-no group individuals (21/23, 91%). Cardiac intervention was performed in 40% of all cases and was statistically significantly associated with better outcomes: 79% of those who were alive at the time when the article was published have received cardiac intervention (p < 0.001). Most of them were valvuloplasty surgeries on mitral and tricuspid valves with a few aortic sleeve placement surgeries. Correspondingly, in cys-no and cys– groups more children reported alive have received cardiac intervention (p < 0.002 vs. p < 0.035).

### Respiratory features

Only information on diaphragm eventration, hernia or relaxation was collected. To note, the largest percentage of diaphragm lesions was in a dominant negative effect group 8/44 (18%): 22% and 17% in cys– and cys-no group, respectively. In contrast, there was only one case of diaphragm lesion observed in the haploinsufficiency group.

## Discussion

In 2007, Faivre et al. established a genotype-phenotype association for classical MFS based on the effects of haploinsufficiency and a dominant negative model on skeletal, ophthalmologic, and cardiac characteristics [50]. Other author analyses revealed that pathogenic variants in exons 24–32 are present in approximately 90% of patients with nMFS, but only in 20% of classic cases [51]. Subsequent studies focused on the clinical relevance and genotype effect on mitral valve phenotype in MFS patients [12, 52]. Understanding how different genotypes impact the development of EOMFS is crucial for accurately predicting the course of the disease and determining the most effective management strategies.

We report a 24-months-old patient with EOMFS and supraventricular tachycardia paroxysm, who presented specific MFS skeletal features. The diagnosis of the syndrome was made relatively late, which indicates either the lack of knowledge about MFS characteristics among healthcare providers or the presence of early onset MFS features that are different from the adult form of the syndrome. Analysis of the cases reported in the literature showed arachnodactyly, reduced elbow extension, pectus deformity, and scoliosis as the most frequently seen skeletal features in EOMFS. As well, we highlight the importance of categorizing patients by their genotype-phenotype profile as it has an impact on clinical decision making. Speaking of mutations spectrum in nMFS compared to the classic MFS form, missense variant overrepresentation is observed. Importantly, PTC mutations are associated with the classical MFS and are rarely found in nMFS, with nonsense pathogenic variants resulting in nMFS not described in the literature [50]. In addition, our study has limitations. First, statistical analysis was limited by disproportionate size of the samples, while incomplete phenotyping, retrospective nature of the study and clinical characteristics reporting bias may have affected conclusions.

Recently, Zarate et al. developed a clinical scoring system for EOMFS, which consider cardiac, systemic and *FBN1* diagnostic features [53]. However, a threshold of 14 points is required if only clinical features are considered and all of them must be present. When comparing results of the present study to those of Zarate et al., the most reported characteristics were arachnodactyly, craniofacial dysmorphism, joint contractures, reduced elbow extension, and lens dislocation/subluxation. If the patient has just one or more but not all the features, a more complex initial clinical evaluation may be required, as rarity of the syndrome and stable general status of the patient may misguide health care specialists and delay final diagnosis. Typically, in such cases, echocardiogram is not performed as cardiovascular system still does not show any signs of impairment. Echocardiogram could be an important cost-effective diagnostic tool in EOMFS diagnosis as the clinical scoring system developed by Zarate et al. may not cover variety of the cases where more comprehensive evaluation may be required.

It is interesting to note the differences in cardiovascular phenotype among the different variants of *FBN1* gene in EOMFS patients, as well the potential impact on clinical management and prognosis. Variants resulting in cysteine alterations and haploinsufficiency, which were associated with higher aortic events risk, highlight the importance of regular cardiovascular monitoring and early intervention planning in these patients [12, 54, 55]. In addition, cys– variants also have the greater prevalence of mitral valve prolapse (MVP) and mitral annular disjunction (MAD). Besides, MAD rises the greater risk for arrhythmic events and is assumed to be a possible cause of sudden death in MFS patients [52]. It poses a risk for our patient, which presented with supraventricular tachycardia and MAD. Close cardiovascular monitoring and family training to recognize paroxysms of arrhythmia are important in these situations. Other studies focusing on EOMFS found out cys-no variants were of intermediate cardiovascular phenotype (61% lifelong risk of surgery or dissection) [12]. In the present study, the better outcomes observed in the cys-no and cys– groups after cardiac intervention suggest that timely and appropriate management can improve outcomes in these patients. However, the higher rates of death in the haploinsufficiency group (87.5%, 7/8), although not statistically significant in the present study, highlights the need for further research and larger studies to better understand the impact of different *FBN1* variants on cardiovascular outcomes in EOMFS patients.

Since diaphragm relaxation is an aggravating circumstance in our case, we reviewed reported cases to better understand the prognosis. Almost all of the patients (7/9) experienced bad outcomes during infancy, while the oldest child was 24 months-old when he passed away suddenly. Four deaths were reported of cardio-respiratory distress syndrome, one of multiple organ failure, one of sudden death and one of respiratory failure after support withdrawal. In most of the other reported cases cardiac or cardiorespiratory insufficiency led to the lethal outcome. Post-mortem analysis of two cases showed emphysematous changes, consolidation and intraalveolar haemorrhage with lung fibrosis. However, majority of the cases had severe diaphragmatic hernia and cardiac lesions with no diaphragmatic relaxation reported, letting us believe it may have better prognosis. All things considered, diaphragm hernia worsen EOMFS patients’ prognosis, but little is known on diaphragm relaxation impact on the disease.

In the literature, pathogenic variants substituting or creating cysteine residuals in *FBN1* gene have a higher probability of ectopia lentis [50]. It seems that the present study’s findings are generally consistent with previous literature regarding the association between pathogenic variants involving cysteine residues in the *FBN1* gene and the likelihood of ectopia lentis. Specifically, both the present study and previous research found that cys + variants have a higher risk of ectopia lentis compared to haploinsufficiency and cys-no groups. However, it should be noted that the cys– group in the present study had a lower frequency of ectopia lentis compared to what has been reported in the literature. It is also worth noting that the cys + group in this study had a relatively small sample size (N = 3), which may limit the conclusions.

Previous studies have shown that haploinsufficiency patients have a significant skeletal system involvement [50]. This was later confirmed by Arnaud et al., who reported a high frequency of severe scoliosis among haploinsufficiency patients (52%), as well as cys– (45%) and cys-no (43%) groups [12]. Our results for cys–, cys-no patients were similar (39% and 48% respectively). However, in the cys + group, we found a higher frequency of scoliosis or thoracolumbar kyphosis (33%) compared to the data of Arnaud et al. (16%). Our findings also revealed a lower frequency of scoliosis among haploinsufficiency patients (12.5%), but higher rates of reduced elbow extension and pectus deformity (75% and 37.5%, respectively), which is consistent with the findings reported by Faivre et al. [50].

In 2016, Maeda et al. reported on two EOMFS patients who survived after the initial diagnosis: 10 and 8 years, respectively [7]. The first patient required three mitral valvuloplasty surgeries and medication therapy, when the second child was successfully developing with medication therapy and foreseen aortic root replacement surgery. Authors suggested that a broader spectrum of EOMFS phenotypes exist and could provide some assistance in treatment decision making. Based on their genotype, those two patients would be assigned to the cys-no group, which has better outcomes with cardiac intervention, a lower risk for ectopia lentis and an intermediate cardiovascular phenotype [12].

Genotype-phenotype categorization could guide physicians in the management of EOMFS. Patients with cys + genotypes should be more intensively screened for ectopia lentis, while haploinsufficiency patients should be closely monitored for cardiovascular state. Speaking of cys– and cys-no patient groups, a possible overlap within phenotype severity and eligibility for surgical intervention based on their genotype should be cautiously investigated in future genotype-phenotype studies, as other authors previously found similar results and suggested to optimise timing and decision making on the need for prophylactic aortic root surgery [54]. All EOMFS patients are at risk for some type of skeletal deformation and should be regularly examined by a pediatric orthopaedic traumatologist.

## Conclusions

This study found that EOMFS patients who were infants and children over one-year-old had more favourable outcomes. Skeletal features such as arachnodactyly, pectus deformity, reduced elbow extension, and scoliosis were common and suggested the need for an echocardiogram. A relationship between better outcomes and performed cardiac intervention in cys– and cys-no groups exists and needs to be further investigated. Our results have important prognostic implications, as different genotype groups may require individualized care and management based on their specific phenotype.

### Electronic supplementary material

Below is the link to the electronic supplementary material.

Supplementary Material 1

## Data Availability

All data generated or analysed during this study are included in this published article or are available from the corresponding author on reasonable request.

## List of Abbreviations

MFS: Marfan syndrome
FBN1: Fibrillin 1
EOMFS: Early–onset Marfan syndrome
nMFS: Neonatal Marfan syndrome
DN: Dominant negative
Cys+: Missense variants that substitute for a cysteine
Cys−: Variants that substitute a cysteine for another amino acid
Cys-no: Variants that do not modify cysteine content
ACMG: American College of Medical Genetics and Genomics
AMP: Association for Molecular Pathology
ECG: Electrocardiogram
CT: Computer tomography
MVP: Mitral valve prolapse
MAD: Mitral annular disjunction
