# Supplementary material for: Genotype-phenotype spectrum and prognosis of early-onset Marfan syndrome
Source: BMC Pediatr. 2023 Oct 28;23:539. doi: 10.1186/s12887-023-04357-8 (PMC10612290; doi:10.1186/s12887-023-04357-8)
Supplement: Supplementary file 1 — Supplementary Material 1 [file 12887_2023_4357_MOESM1_ESM.docx]

**Supplementary Table 1:** The *FBN1* pathogenic variants of the reported cases.

| **References** | **Nucleotide and protein change as reported in the reference literature** | **Nucleotide and protein change, dbSNP Reference SNP according to HGVS (NM_000138.5)** | **Group** |
| --- | --- | --- | --- |
| Rosendaal L et al., 2011 [16] | c.3396delA, p.(Glu1133ArgfsX29) | c.3396del, p.(Glu1133ArgfsTer29) | Hap |
| Hanseus K et al., 1995 [49] | cDNA 389_514del | NA | Hap |
| Shinawi M et al., 2005 [47] | IVS31-2A>G | c.3713-2A>G, p.(?) | Hap |
| Booms P et al., 1999 [6] | IVS31+1G>A | c.3838+1G>A, p.(?) | Hap |
| Maeda J et al., 2016 [7] | c.IVS29+1G>A | c.3589+1G>A, p.(?) | Hap |
| Maeda J et al., 2016 [7] | c.IVS30+1G>A | c.3712+1G>A, p.(?) | Hap |
| Yoon SH et al., 2021 [44] | c.3964+1G>T | c.3964+1G>T, p.(?) | Hap |
| Barnett CP et al., 2010 [23] | c.3838+1G>A | c.3838+1G>A, p.(?) | Hap |
| Ardhanari M et al., 2019 [15] | c.3557A>G, p.(Tyr1186Cys) | c.3557A>G, p.(Tyr1186Cys), rs1555398511 | Cys+ |
| Motonaga T et al., 2022 [17] | c.3379G>T, p.(Gly1127Cys) | c.3379G>T, p.(Gly1127Cys) | Cys+ |
| Milewicz MD et al., 1994 [18] | 3-bp insertion between nucleotides 480 and 481 or 481 and 482 (or nucleotides 3174 and 3175 or 3175 and 3176) | NA | Cys+ |
| This report | c.3661T>C, p.(Cys1221Arg) | c.3661T>C, p.(Cys1221Arg) | Cys - |
| Ardhanari M et al., 2019 [15] | c.3661T>C, p.(Cys1221Arg) | c.3661T>C, p.(Cys1221Arg) | Cys - |
| Peng Q et al., 2016 [19] | c.3331T>C, p.(Cys1111Arg) | c.3331T>C, p.(Cys1111Arg) | Cys - |
| Summers KM et al.,2005 [20] | c.3131G>A, C1044Y | c.3131G>A, p.(Cys1044Tyr), rs730880100 | Cys - |
| Putnam EA et al., 1996 [8] | 3349T>C, C1117G | c.3349T>C, p.(Cys1117Arg) | Cys - |
| Otero Luna A et al., 2021 [21] | c.3257G>C, p.C1086S | c.3257G>C, p.(Cys1086Ser) | Cys - |
| Kawamura J et al., 2022 [22] | c.3706T>C, p.(Cys1236Arg) | c.3706T>C, p.(Cys1236Arg), rs1555398380 | Cys - |
| Barnett CP et al., 2010 [23] | c.3202T>G, p.C1068G | c.3202T>G, p.(Cys1068Gly), rs1293095681 | Cys - |
| Kochilas L et al., 2008 [24] | c.3256T>C, C1086R | c.3256T>C, p.(Cys1086Arg), rs1555398637 | Cys - |
| Derbent M et al., 2007 [25] | c.3602G>A, p.(Cys1201Tyr) | c.3602G>A, p.(Cys1201Tyr), rs1555398413 | Cys - |
| Tekin M et al., 2007 [26] | c.3257G>A, p.(Cys1086Tyr) | c.3257G>A, p.(Cys1086Tyr), rs137854484 | Cys - |
| Sutherell J et al., 2007 [27] | c.3256T>C, p.(Cys1086Arg) | c.3256T>C, p.(Cys1086Arg), rs1555398637 | Cys - |
| Nazarali S et al., 2017 [45] | Deletion of exons 32-37 | c.(3838+1_3839−1)_ (4582+1_4583−1)del | Cys - |
| Apitz C et al., 2010 [46] | Deletion of exons 24-26 | c.(2728+1_2729−1)_ (3208+1_3209−1)del | Cys - |
| Elgaz S et al., 2022 [48] | c.(5065+1_5066−1)_ (5545+1_5546−1)del  in-frame deletion of exons 42–45 | c.(5065+1_5066−1)_ (5545+1_5546−1)del | Cys - |
| Whitelaw CM et al., 2004 [28] | c.3202T>C and c.3204C>G (in cis) p.(Cys1068Arg) | c.3202_3204delinsCGG, p.(Cys1068Arg) | Cys - |
| Revencu N et al., 2003 [29] | c.3165T>G, C1055W | c.3165T>G, p.(Cys1055Trp), rs1060501040 | Cys - |
| Putnam EA et al., 1996 [8] | 3157T>C, C1053R | c.3157T>C, p.(Cys1053Arg) | Cys - |
| Mbwasi RM et al., 2022 [30] | NM_000138.3:c.7606G>A | c.7606G>A, p.(Gly2536Arg), rs397515854 | Cys-no |
| Willis BR et al., 2020 [31] | NM_000138.4: c.3037G>A, p.G1013R | c.3037G>A, p.(Gly1013Arg), rs140593 | Cys-no |
| Ardhanari M et al., 2019 [15] | c.3037G>A, p.(Gly1013Arg) | c.3037G>A, p.(Gly1013Arg), rs140593 | Cys-no |
| Maeda J et al., 2016 [7] | c.3677G>T, p.(Gly1226Val) | c.3677G>T, p.(Gly1226Val), rs1555398387 | Cys-no |
| Sureka D et al., 2014 [32] | c.3143T>C, I1048T | c.3143T>C, p.(Ile1048Thr), rs1555398673 | Cys-no |
| Sureka D et al., 2014 [32] | c.3143T>C, I1048T | c.3143T>C, p.(Ile1048Thr), rs1555398673 | Cys-no |
| Sureka D et al., 2014 [32] | c.3143T>C, I1048T | c.3143T>C, p.(Ile1048Thr), rs1555398673 | Cys-no |
| Wang M et al., 1997 [41] | c.3128A>G, K1043R | c.3128A>G, p.(Lys1043Arg), rs137854472 | Cys-no |
| Putnam EA et al., 1996 [8] | 3215A>G, D1072G | c.3215A>G, p.(Asp1072Gly) | Cys-no |
| Wadia T et al., 2022 [34] | c.3143T>C | c.3143T>C, p.(Ile1048Thr), rs1555398673 | Cys-no |
| Carande EJ et al., 2017 [35] | c.3143T>C | c.3143T>C, p.(Ile1048Thr), rs1555398673 | Cys-no |
| Kuruvilla SE et al., 2019 [36] | c.3038G>C | c.3038G>C, p.(Gly1013Ala) | Cys-no |
| Tognato E et al., 2019 [37] | c.3143T>C, p.(Ile1048Thr) | c.3143T>C, p.(Ile1048Thr), rs1555398673 | Cys-no |
| Heo JS et al., 2016 [38] | c.3217G>A, E1073K | c.3217G>A, p.(Glu1073Lys), rs137854478 | Cys-no |
| Maeda J et al., 2016 [7] | c.3217G>A, p.(Glu1073Lys) | c.3217G>A, p.(Glu1073Lys), rs137854478 | Cys-no |
| Maeda J et al., 2016 [7] | c.3217G>A, p.(Glu1073Lys) | c.3217G>A, p.(Glu1073Lys), rs137854478 | Cys-no |
| Sureka D et al., 2014 [32] | c.3143T>C, I1048T | c.3143T>C, p.(Ile1048Thr), rs1555398673 | Cys-no |
| Chao SC et al., 2010 [39] | c.3208G>C | c.3208G>C, p.(Asp1070His) | Cys-no |
| Jacobs AM et al., 2002 [40] | T3276C, I1048T | c.3143T>C, p.(Ile1048Thr) | Cys-no |
| Wang M et al., 1997 [33, 41] | c.3391A>T, N1131Y | c.3391A>T, p.(Asn1131Tyr), rs137854473 | Cys-no |
| Lönnqvist L et al., 1996 [42] | T3143C, I1048T | c.3143T>C, p.(Ile1048Thr), rs1555398673 | Cys-no |
| Bresters D et al., 1999 [43] | 3276T→C, 1048Ile→Thr | c.3143T>C, p.(Ile1048Thr), rs1555398673 | Cys-no |
| Putnam EA et al., 1996 [8] | 3141_3143del, Del I1048 | c.3141_3143del, p.(Ile1048del) | Cys-no |

Variants that substitute a cysteine for another amino acid (Cys-); missense variants that substitute for a cysteine (Cys+); variants that do not modify cysteine content (Cys-no); haploinsufficiency (Hap); not applicable (NA).
